# Dynamic patterns of electroosmosis peristaltic flow of a Bingham fluid model in a complex wavy microchannel

**DOI:** 10.1038/s41598-023-35410-2

**Published:** 2023-05-29

**Authors:** H. A. Hosham, Esraa N. Thabet, A. M. Abd-Alla, S. M. M. El-Kabeir

**Affiliations:** 1 Department of Mathematics, Faculty of Science, Al-Azhar University, Assiut, 71524 Egypt; 2 Department of Mathematics, Faculty of Science, Aswan University, Aswan, Egypt; 3 Department of Mathematics, Faculty of Science, Sohag University, Sohag, Egypt

**Keywords:** Applied mathematics, Computational science

## Abstract

The purpose of this paper is to present a rigorous analysis of streamline patterns and their bifurcation to a viscoplastic Bingham fluid model that involves heat and mass transfer in an electroosmotic flow through a complex wavy microchannel. The Bingham fluid act as a solid medium in the core layer, which divides the channel into three distinct sections utilized to model the problem as a switched dynamical system between these zones. To track multiple steady states (stagnation points) and related trapping phenomena, we perform both analytical and numerical bifurcation analysis of each subsystem with respect to different physical effects such as electrical double layer thickness and Helmholtz-Smoluchowski velocity. The key feature of the technique presented here is its ability to reveal the peristaltic transport characteristics of the Bingham fluid model in the presence or absence of symmetric flow properties. The primary novelty here is the ability to regulate the location and stability of the equilibrium points in the domain of interest. This leads to the detection of global bifurcations that reflect important dynamic elements of the model. Our results highlighted a new category of complex behavior that controls transitions between qualitatively different transport mechanisms, as well as a class of non-classical trapping phenomena.

## Introduction

The use of bio-inspired materials has been increasingly prevalent in modern engineering model improvements. In order to update traditional engineering systems and attain previously unheard-of levels of endurance and performance, a number of intricate processes have been developed using biological systems. One such process is peristaltic movement. The contraction and expansion of a fluid-filled, flexible, tubular structure is known as peristalsis. Peristalsis has a wide variety of uses, and there are numerous researches regarding it in the literature^1–5^ Numerous physiological processes, such as bile transfer in a bile duct, semen movement in the vas deferens, and blood flow in tiny capillaries are governed by this idea. Both blood pumps and heart-lung devices operate on a similar premise^6,7^.

Boosting heat transfer inside a channel is crucial to create more compact heat exchangers, which are used in a variety of engineering applications like cooling for electronic devices, air conditioning equipment, and ocean thermal energy conversion technologies. For scientists and engineers, the creation of these technologies is of the utmost importance^8^.

Microchannel procedures produce electroosmotic fluxes, and chemical separation is used in a variety of biotechnology applications^9–11^. The field of electrokinetic transfer has seen a boom in modern fluid mechanics. The interaction of electrolytic fluids with external electric fields, either static or alternating, is explored experimentally and analytically. Charge distributions, wetted surfaces, zeta potentials, and electric double layers are just a few of the fascinating phenomena it exhibits. Electrokinetic encompasses a wide range of phenomena, including electroosmosis, electrophoresis, and diffusiophoresis (where chemical gradients are important). Electroosmosis in narrow micro-vessels^12^. Diversified bio microfluidics systems^13^.

Complex peristaltic pumping or complex boundary wall wavy patterns are two more significant peristaltic pumping occurrences. In industry, complicated pumping phenomena are employed to increase the effectiveness of micro and nano pumps, particularly in the medical field. Electroosmotic flow of pseudoplastic nano liquids via peristaltic pumping biomimetic propulsion is causing complex wavy, curving surfaces to be studied. Complex wavy channel with MHD effects^14–16^. electro-magneto-hydrodynamics^17^. Heat and mass transfer in complicated, wavy microchannels of microvascular blood flow^18^, and some literatures were found^19–24^.

A branch of non-Newtonian fluids known as viscoplastic materials has a yield stress threshold for the applied stress. The material deforms as a viscous fluid for applied stresses greater than the yield stress; for applied forces less than the yield stress, the material behaves as a rigid solid. The phrase “viscoplastic materials” in this context often refers to substances that solely have viscous and plastic qualities. The first and simplest model is the Bingham fluid^25^. Numerous engineering applications, including petroleum engineering, the discharge of groundwater into aquifers, and MHD generators, require a thorough understanding of Bingham fluid flow^26–28^.

When parameters are altered or when variables and processes interact, dynamic systems theory outlines methods for analysing stability and changes in a system’s overall structure^29–32^. With the help of this method, you may identify the dynamics of fluid physical events and gain a better knowledge of the behaviour of the system as a whole. Regarding this, stability and bifurcation theory for streamline patterns has recently been created in order to offer critical insights for regulating and recognizing fluid transport mechanisms in specific classes of fluid-mechanical systems^33,34^. There have been several attempts to discuss the structural bifurcations and associated stagnation spots for two-dimensional incompressible flow, with the flow supposedly being explicitly represented by a polynomial form^35,36^. The streamline patterns and bifurcations around the stagnation (equilibrium) regions of peristaltic flow of various biological fluids are explored^37–39^, they determined the important qualitative aspects of the peristaltic flow, such as “bolus” (or “trap”) on the assumption that the flow motion shape was produced by a uniform channel.

In this paper, we focus on the topology of streamlines in a viscoplastic Bingham fluid model that includes heat and mass transfer in an electroosmotic flow through a complex wavy microchannel, especially on issues about bifurcations and stability, which are used to predict flow phenomena in streamline patterns when physical parameters are changed. The Bingham fluid (which exhibits the dual behaviour of a fluid and a solid) channel has one plug region and two distinct non-plug regions in the wave frame. Because the flow behavior in the plug region is constant, the nonlinear switched dynamical system is formed by connecting the obtained expression of the stream function with velocity fields in two distinct non-plug regions. The equilibrium points of each subsystem are calculated analytically and numerically, and a bifurcation analysis of these points is performed to show how topological characteristics change as one or more physical parameters are changed. The equilibrium point is classified as admissible or virtual depending on whether it is within or outside the domain of validity. The transition of virtual to admissible equilibrium points and the possibility of bifurcation in each domain independently are obtained. A heteroclinic connection is formed when a streamline connects two or more saddle-points. The use of heteroclinic connections in a subsystem or a full system has the advantage of completely trapping the flow between the orbits of the connection. If a heteroclinic connection exists, the distance between two saddle points is used to determine the minimum (or maximum) trapping zone limits. The bifurcation results are used to explain the effect of various physical parameters on fluid flow behaviors, such as flow rate, plug region size, electrical double layer thickness, and Helmholtz-Smoluchowski velocity.

The paper is structured as follows: Sect. “Mathematical configuration” explains the mathematical formulation of the viscoplastic Bingham fluid model and its potential applications. Section “Explicit analytical solutions” provides closed form solutions for the stream and potential functions, as well as particle concentration and temperature distribution. Section “Bifurcations of equilibria and nonlinear behavior” describes the details of the formed dynamical system, and analytical formulas for the position and nature of the equilibrium points in a specific scenario are obtained. Section “Results and discussion” describes the numerical bifurcation analysis approach that is used to analyze the model’s actual scenario. Section “Conclusions” discusses the impact of different physical parameters on fluid flow behaviors and presents the results of numerical bifurcation analysis.

## Mathematical configuration

### Geometric structure

**Figure 1:**
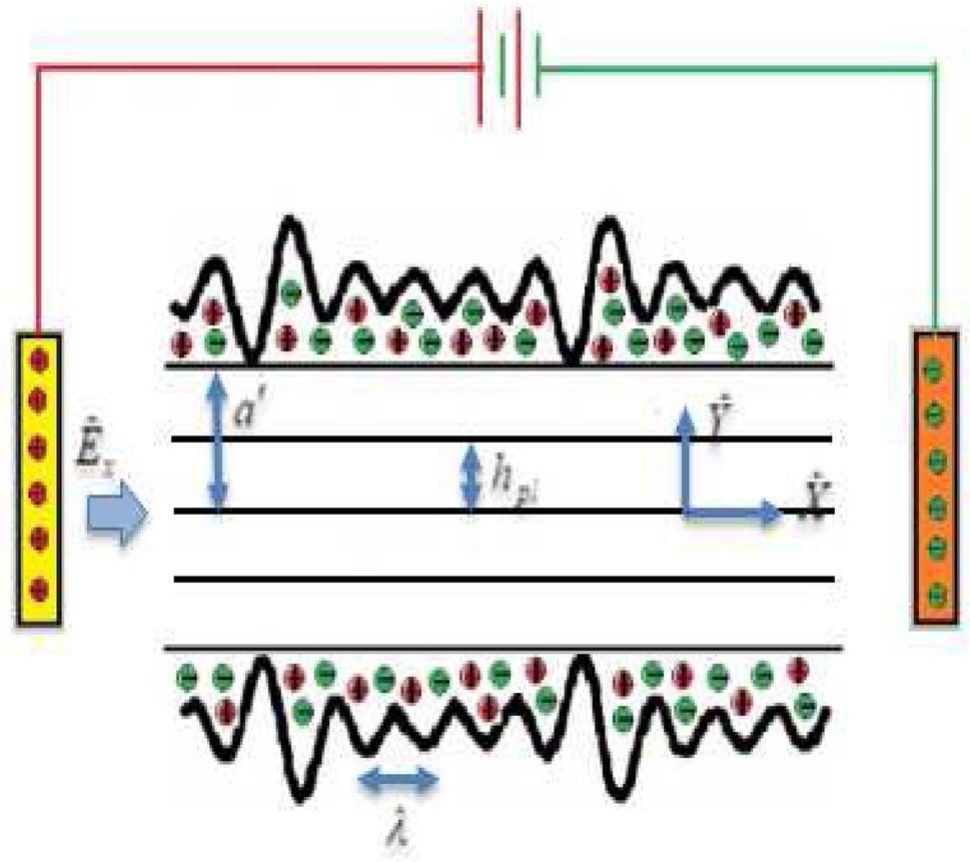
The geometric representation of electroosmosis flow is depicted in a microchannel resembling a wave.

The electroosmosis peristaltic transport flow is described using a complex wavy two-dimensional microchannel. An externally supplied electric field is used through this channel to adjust the electroosmotic flow of an aqueous ionic solution in a non-Newtonian fluid (Bingham viscoplastic fluid), see Fig. 1. Viscous dissipation and joule heating are also considered. A mathematical model of wall deformation^17,18^ is shown below.

1 $$\begin{aligned} \widehat{H}\left( \widehat{X},\widehat{t}\right) = \acute{a}+\sum _{i=1}^{m} \widehat{\varepsilon _{i}} \sin \left( \frac{2 i \pi }{\lambda } \left( \widehat{X}-c \widehat{t}\right) \right) , ~~~~ m\in {Z^{+}}. \end{aligned}$$

where $\widehat{\varepsilon _{i}}$ needs to fulfil the condition $\sum _{i=1}^{m} \widehat{\varepsilon _{i}}\le \acute{a}$.

Bingham fluid is a viscoplastic fluid, a type of non-Newtonian fluid in which the flow field is divided into two regions: an un-yielded zone in which the fluid is at rest or undergoes stiff motion, and a yielded zone in which the fluid flows like a viscous liquid. In the un-yielded zone, the second invariant of the extra stress tensor is less than or equal to the yield stress and a constitutive relation is undefined. In the yielded region, this invariant exceeds the yield stress and a constitutive relation exists for the extra stress tensor. Thus, the location and shape of the yield surface(s), i.e. the interface between these two sets, is also a part of the solution of flow problems of such fluids. Viscoplastic fluids occur in various chemical, metal, and food industries, e.g., margarine, mayonnaise and ketchup. The constitutive equation of an incompressible Bingham fluid is based on the assumption that the fluid remains at rest or moves as a rigid body if the second invariant of the extra stress tensor $S_{xy}$ is less than or equal to the yield stress $S_0$.

The constitutive equation^18,25^ that is required to describe a Bingham model is:

2 $$\begin{aligned} {\widehat{S}}_{x y}&= \left\{ \begin{array}{cc} \widehat{S_{0}}+\mu \widehat{\dot{\gamma }}; &{} \widehat{S}_{x y} \ge \widehat{S}_{0}, \\ \widehat{S_{0}};&{} \widehat{S}_{x y}< \widehat{S}_{0}, \end{array} \right. \end{aligned}$$

where $\widehat{S_{0}}$ represents yield stress and $\widehat{\dot{\gamma }}$ represents the rate of strain tensor. As a direct consequence, the Bingham fluids act as a solid medium in the core layer.

### Bingham fluid applications

The hypothetical viscous fluid has a yield strength that needs to be exceeded before it may flow. A lava channel, for example, is defined as a stream of flowing lava contained within zones of static (i.e., solid and motionless) lava or lava levees. Levees may not exist in the original channel until the parental flow settles over what forms the channel and produces natural levees. Therefore, lava behaves as a multiphase non-Newtonian fluid, for example see^40^. In this context, most lava flows are obvious applications of Bingham fluids. additionally, Bingham Fluid can be designed as the interaction of blood’s non-Newtonian nature and its flow through arteries, such as microvascular blood flow through a complex wavy microchannel, see^10,18^.

### Governing equations

The governing equations that serve as guiding principles for electroosmotic fluid flow^18^ are given by:

3 $$\begin{aligned}{} & {} \frac{\partial \widehat{U}}{\partial \widehat{X}}+ \frac{\partial \widehat{V}}{\partial \widehat{Y}}= 0, \end{aligned}$$

4 $$\begin{aligned}{} & {} \rho D_{e}\widehat{U}= -\frac{\partial \widehat{P}}{\partial {\widehat{X}}}+\frac{\partial \widehat{S}_{\widehat{X}\widehat{X}}}{\partial \widehat{X}}+\frac{\partial \widehat{S}_{\widehat{X}\widehat{Y}}}{\partial \widehat{Y}}+\rho _{e}\widehat{E_{x}}, \end{aligned}$$

5 $$\begin{aligned} \rho{} & {} D_{e}\widehat{V}=-\frac{\partial \widehat{P}}{\partial \widehat{Y}}+\frac{\partial \widehat{S}_{\widehat{X}\widehat{Y}}}{\partial \widehat{X}}+\frac{\partial \widehat{S}_{\widehat{Y}\widehat{Y}}}{\partial \widehat{Y}}+\rho _{e}\widehat{E_{x}}, \end{aligned}$$

6 $$\begin{aligned}{} & {} \rho C_{p} D_{e}\widehat{T}=K \left( \frac{\partial ^2 \widehat{T}}{\partial {\widehat{X}}^2}+\frac{\partial ^2 \widehat{T}}{\partial {\widehat{Y}}^2}\right) +\acute{S}+ \sigma \left( \widehat{E}.\widehat{E} \right) , \end{aligned}$$

7 $$\begin{aligned}{} & {} D_{e}\widehat{C}=D_{m}\left( \frac{\partial ^2 \widehat{C}}{\partial {\widehat{X}}^{2}}+\frac{\partial ^2 \widehat{C}}{\partial {\widehat{Y}}^{2}}\right) +K_{\acute{r}}\widehat{C}. \end{aligned}$$

where, $D_{e}=\frac{\partial }{\partial \widehat{t}}+\widehat{U}\frac{\partial }{\partial \widehat{X}}+\widehat{V}\frac{\partial }{\partial \widehat{Y}}$ is the differential operator. The variables and parameters used in the preceding system are defined in List of symbols section. Due to the presence of an electric double layer (EDL) in the microchannel, the electric potential is calculated using the Poisson equation^12^, which can be expressed as:

8 $$\begin{aligned} \nabla ^2\widehat{\Theta }=-\frac{\rho _{e}}{\acute{\varepsilon }}, \end{aligned}$$

such that $$\rho _{e}=ze\left( \widehat{n}_{+}-\widehat{n}_{-}\right) $$.

In this context, the Nernst-Planck equation for ionic number distribution is utilized to assess potential distribution as follows:

9 $$\begin{aligned} \begin{aligned} D_{e}\widehat{n}_{\pm }= \frac{D_{m}ze}{k_{B}T_{a}} \left[ \frac{\partial }{\partial \widehat{X}}\left( \widehat{n}_{\pm }\frac{\partial \widehat{\Theta }}{\partial \widehat{X}}\right) +\frac{\partial }{\partial \widehat{Y}}\left( \widehat{n}_{\pm }\frac{\partial \widehat{\Theta }}{\partial \widehat{Y}}\right) \right] +D_{m}\left( \frac{\partial ^2 \widehat{n}_{\pm }}{\partial \widehat{X}^2}+\frac{\partial ^2 \widehat{n}_{\pm }}{\partial \widehat{Y}^2}\right) . \end{aligned} \end{aligned}$$

We introduce the following set of non-dimensional variables and parameters to help get explicit analytical solutions to the governing equations.

10 $$\begin{aligned} x&=\frac{\widehat{x}}{\lambda }, ~~ y=\frac{\widehat{y}}{\acute{a}}, ~~ t=\frac{\widehat{t}c}{\lambda }, ~~ u=\frac{\widehat{u}}{c}, ~~ \nonumber \\ v&=\frac{\widehat{v}}{\delta c}, ~~ h=\frac{\widehat{H}}{\acute{a}}, ~~~\varepsilon =\frac{\widehat{\varepsilon }}{\acute{a}}, \nonumber ,\, Re=\frac{\rho c \acute{a}}{\mu }, ~~~ \delta =\frac{\acute{a}}{\lambda }, ~~ S_{x y}=\frac{\widehat{S}_{\widehat{X} \widehat{Y}}\acute{a}}{\mu c}, \\ S_{0}&=\frac{\widehat{S}_{0}\acute{a}}{\mu c}, ~~ p=\frac{\widehat{P}\acute{a}^2}{\mu \lambda c}, \nonumber \\ \theta&=\frac{T-T{s}}{T_{e}-T_{s}}, ~~\phi =\frac{C-C{s}}{C_{e}-C_{s}}, ~~ \Theta =\frac{ze\widehat{\Theta }}{k_{B}T_{a}}, ~~ \acute{S}=s_{xy} \frac{\partial u}{\partial y}, \nonumber \\ \, \acute{G}&=\frac{\sigma \widehat{E_{x}^2}\acute{a}^2}{{K}\left( T_{e}-T_{s}\right) }, ~~ Br=\frac{\mu c^2}{K\left( T_{e}-T_{s}\right) }, ~~ k_{r}=\frac{{K}_{r}\acute{a}^2}{\nu }, \nonumber \\ S_c&=\frac{\nu }{D_{m}} , ~~ N_c=\frac{C_{s}}{\left( C_{e}-C_{s}\right) }, ~~ \acute{u}_{e}= -\frac{E_{x}\rho \acute{\varepsilon }}{\mu c}, ~~ n=\frac{\widehat{n}}{n_{0}} \end{aligned}$$

## Explicit analytical solutions

Debye-Huckel modifies the Poisson equation as follows:

11 $$\begin{aligned} \frac{\partial ^2\Theta }{\partial y^2} = -\frac{D_{l}}{2}\left( n_{+}-n_{-}\right) . \end{aligned}$$

where, $D_{l}=a ze\sqrt{\frac{2n_{0}}{\acute{\varepsilon } T_{a} k_{B}}}$. Additionally, the dimensionless quantities (10) allow for a reduction of the Nernst-Planck Eq. (9) to establish the ionic distribution as follows:

12 $$\begin{aligned} \frac{\partial ^2 {n}_{\pm }}{\partial y^2}\pm \frac{\partial }{\partial y}\left( n_{\pm }\frac{\partial \Theta }{\partial y}\right) =0. \end{aligned}$$

Solving the above equation with the associated conditions ($\frac{\partial n_{\pm }}{\partial y}=0$  at   $\frac{\partial \Theta }{\partial y}=0$), and ($n_{\pm }=1 ~at ~\Theta =0$) we obtain:

13 $$\begin{aligned} n_{\pm }=\exp {\left( \mp \Theta \right) }. \end{aligned}$$

Thus, the Eq. (11) becomes

14 $$\begin{aligned} \frac{\partial ^2\Theta }{\partial y^2} = {D_{l}}^2\sinh \left( {\Theta }\right) . \end{aligned}$$

The above equation can be linearized using low-zeta potential approximation (i.e, $\sinh \left( \Theta \right) \simeq \Theta$), as:

15 $$\begin{aligned} \frac{\partial ^2\Theta }{\partial y^2} = {D_{l}}^2 \Theta . \end{aligned}$$

The potential function is obtained as a explicit solution of Eq. (15) subject to the boundary conditions ($0=\frac{\partial \Theta }{\partial y}\mid _{y=0}$ and $1=\Theta \mid _{y=h}$) as:

16 $$\begin{aligned} \Theta =\frac{\cosh \left( {D_{l}y}\right) }{\cosh \left( {D_{l}h}\right) }. \end{aligned}$$

The governing Eqs. (1)-(7) for electroosmotic fluid flow are reduced to non-dimensional forms using the dimensionless quantities (10), lubrication theory, low Reynolds, and large wavelength approximations, as follows:

17 $$\begin{aligned}{} & {} h\left( x,t\right) = 1+\sum _{i=1}^{m} \varepsilon _{i} \sin \left( 2 i \pi \left( x-t\right) \right) , ~ \sum _{i=1}^{m} \varepsilon _{i}\le 1 \end{aligned}$$

18 $$\begin{aligned}{} & {} s_{x y} = \left\{ \begin{array}{cc} S_{0}+\frac{\partial u}{\partial y}, &{} s_{x y} \ge s_{0}, \\ s_{0},\text {implicit that } \frac{\partial u}{\partial y} =0 &{} s_{x y}<s_{0}, \end{array} \right. \end{aligned}$$

19 $$\begin{aligned}{} & {} \frac{\partial u}{\partial x}+ \frac{\partial v}{\partial y}=0, \end{aligned}$$

20 $$\begin{aligned}{} & {} \frac{\partial s_{x y}}{\partial y}= \frac{\partial p}{\partial x} - D_{l}^2 \acute{u}_{e}\frac{\cosh \left( {D_{l}y}\right) }{\cosh \left( {D_{l}h}\right) }, \end{aligned}$$

21 $$\begin{aligned}{} & {} \frac{\partial p}{\partial y} =0, \end{aligned}$$

22 $$\begin{aligned}{} & {} \frac{\partial ^2\theta }{\partial y^2} =-\acute{G}-Br \acute{S}, \end{aligned}$$

23 $$\begin{aligned}{} & {} \frac{\partial ^2\phi }{\partial y^2} = k_r S_c \left( \phi +N_d\right) . \end{aligned}$$

Physical boundary conditions for temperature, concentration, and velocity are imposed as^18^:

24 $$\begin{aligned} \begin{aligned} s_{x y}\mid _{y=0}&=u\mid _{y=h}=\frac{\partial \theta }{\partial y}\mid _{y=h_{pl}}=0, ~~ s_{x y}\mid _{y=h_{pl}}=s_{0}\\ \theta \mid _{y=h}&=\phi \mid _{y=h_{pl}}=1, ~~ \phi \mid _{y=h}=0. \end{aligned} \end{aligned}$$

The axial velocity is determined by solving the Eq. (20) with boundary conditions (24) as follows:

25 $$\begin{aligned} \begin{aligned} u&= \acute{u}_{e}\left( 1-\frac{\cosh \left( {D_{l}y}\right) }{\cosh \left( {D_{l}h}\right) }+D_{l}\left( y-h)\right) \frac{\sinh \left( {D_{l}h_{pl}}\right) }{\cosh \left( {D_{l}h}\right) }\right) \\&\quad +\frac{1}{2} \frac{\partial p}{\partial x}\left( y^2-h^2\right) -h_{pl}\frac{\partial p}{\partial x}\left( y-h)\right) , ~~~~ h_{pl}\le y \le h \end{aligned} \end{aligned}$$

The solution for the normalized temperature distribution is found by solving Eq. (22) and using the associated boundary conditions (24) as follows:

26 $$\begin{aligned} \theta =C_4 y^4+C_3 y^3+ C_2 y^2 +C_1 y+ C_0, \end{aligned}$$

where (to simplify, use $D_p=\frac{\partial p}{\partial x}$ ),

$$\begin{aligned} \begin{aligned} C_0=&\,\frac{1}{12 {D_{{l}}}^{2} \left( \cosh \left( hD_{{l}} \right) \right) ^{2}} \left( \left( \left( \left( 3\,{\acute{u}_{e}}^{2}+h{D_{{p}}}^{2} \left( {h}^{3}-2\,{h}^{2}h_{{{pl}}}+2\,{h_{{{pl}}}}^{3} \right) \right) {Br} \right. \right. \right. \\&\left. \left. \left. \quad +12+6\,\acute{G}{h}^{2}-12\,h \acute{G} h_{{{pl}}} \right) {D_{{l}}}^{2}+48\,{Br}\,D_{{p}}\acute{u}_{e} \right) \left( \cosh \left( hD_{{l}} \right) \right) ^{2} \right. \\&\quad +2\,{Br}\,D_{{p}} \left( hD_{{l}} \left( -12+ \left( -3\,{h_{{{pl}}}}^{2}+{h}^{2} \right) {D_{{l}}}^{2} \right) \sinh \left( h_{{{pl}}}D_{{l}} \right) \right. \\&\quad \left. -12\,D_{{l}} \left( h -0.5\,h_{{{pl}}}\right) \sinh \left( hD_{{l}} \right) -6\,D_{{l}}\sinh \left( yD_{{l}} \right) h_{{ {pl}}} \right. \\&\quad \left. +6\,{D_{{l}}}^{2}h_{{{pl}}}\cosh \left( h_{{{pl}}}D_ {{l}} \right) h -24\,\cosh \left( yD_{{l}} \right) \right) \acute{u}_{e}\, \cosh \left( hD_{{l}} \right) \\&\quad -3\,{D_{{l}}}^{2}{Br}\, \left( \left( -2\,hD_{{l}}\cosh \left( h_{{{pl}}}D_{{l}} \right) -4\, \sinh \left( yD_{{l}} \right) \right. \right. \\&\quad \left. \left. \left. +4\, \sinh \left( hD_{{l}} \right) \right) \sinh \left( h_{{{pl}}}D_{{l}} \right) + \left( \cosh \left( yD_{{l}} \right) \right) ^{2}+h{D_{{l}}}^{2} \left( -2\,h_{{{ pl}}}+h \right) \right) {\acute{u}_{e}}^{2}\right) ,\\ C_1=&\,\frac{1}{6 D_{{l}} \left( \cosh \left( hD_{{l}} \right) \right) ^{2}} \left( 6\,h_{{pl}} \left( -1/6\,{D_{{p}}}^{2}Br\,{h_ {{pl}}}^{2}+\acute{G} \right) D_{{l}} \left( \cosh \left( hD_{{l}} \right) \right) ^{2}\right. \\&\left. \quad + 3\,Br\,D_{{p}}{\acute{u}_{e}}\, \left( \left( 4 +{D_{{l}}}^{2}{h_{{pl}}}^{2} \right) \sinh \left( h_{{pl}} D_{{l}} \right) \right. \right. \\&\quad \left. \left. +4\,\sinh \left( yD_{{l}} \right) -2\,D_{{l}}\cosh \left( h_{{pl}}D_{{l}} \right) h_{{pl}} \right) \cosh \left( hD_{{l}} \right) \right. \\&\quad \left. -3\,{D_{{l}}}^{2}Br\,{{\acute{u}_{e}}}^{2} \left( \cosh \left( h_{{pl}}D_{{l}} \right) \sinh \left( h_{{{ pl}}}D_{{l}} \right) +h_{{pl}}D_{{l}} \right) \right) \\ C_2=&\,{\frac{-2\,G \cosh ^{2}\left( h D_{l}\right) +{D_{l}}^{2 }{Br}\,{{\acute{u}_{e}}}^{2}}{4 \left( \cosh \left( hD_{l} \right) \right) ^{ 2}}}, ~~ C_4=-\frac{1}{12} Br D_p,\\ C_3=&\,{\frac{D_p{ Br}\, \left( D_p h_{{{pl }}}\cosh \left( hD_{l} \right) -D_{l}\sinh \left( h_{{pl}}D_{l} \right) { \acute{u}_{e}} \right) }{6 \cosh \left( hD_{l} \right) }}. \end{aligned} \end{aligned}$$

Further solving Eq. (23) with the subject to the boundary conditions given Eq.(24) yields,

$$\begin{aligned} \begin{aligned} \phi&=\frac{1}{\alpha _{{1}}\sinh \left( \sqrt{\alpha _ {{1}}} \left( h -h_{pl} \right) \right) }\left( -\alpha _{{2}}\sinh \left( \sqrt{\alpha _{{1}}} \left( h -h_{pl} \right) \right) + \left( \alpha _{{1}}\right. \right. \\&\quad \left. \left. +\alpha _{{2}} \right) \sinh \left( \sqrt{\alpha _{{1}}} \left( h-y \right) \right) +\alpha _{{2}}\sinh \left( \sqrt{\alpha _{{1}}} \left( y-h_{pl} \right) \right) \right) . \end{aligned} \end{aligned}$$

where $$\alpha _1=k_r S_c$$ and $$\alpha _2=k_r S_c N_d$$.

## Bifurcations of equilibria and nonlinear behavior

The main goal of this section is to describe and control the nonlinear behavior of all flow modes using dynamical system theory and state space simulations. The equations of motion of individual fluid particles in a two-dimensional flow can be written in classical form, with the stream function acting as the Hamiltonian switched dynamical system. The following regions are defined based on the problem formulation and stage configuration flow.

- Upper region of non-plug flow $\Sigma _1:=\{\left( x,y\right) \in \mathbb {R}^2 \mid h_{pl}\le y\le h\left( x,y\right) \}$.
- Plug flow region $\Sigma _2:=\{\left( x,y\right) \in \mathbb {R}^2 \mid 0\le y\le h_{pl}\}$.
- Lower region of no-plug flow $\Sigma _3:=\{\left( x,y\right) \in \mathbb {R}^2 \mid y\le 0\}$.

Thus, for the vector $\xi \in \mathbb {R}^2$, the switching lines are defined by $y=h_{pl}$ and $y=0$ and the vector fields $f_i(\xi ,\vartheta )$ are smooth functions on the corresponding region $\Sigma _i$, i=1,2,3. The switching system is characterized as:

27 $$\begin{aligned} \dot{\xi }&= \left\{ \begin{array}{cc} f_1 (\xi ,\vartheta ), &{} \xi \in \Sigma _1 , \\ f_2 (\xi ,\vartheta ), &{} \xi \in \Sigma _2, \\ f_3 (\xi ,\vartheta ), &{} \xi \in \Sigma _3, \end{array} \right. \end{aligned}$$

where $\vartheta \in \mathbb {R}^d$ is a *d*-dimensional parameter space and $\xi =\left( x,y\right) ^T\in \mathbb {R}^2$. It should be emphasized that no dynamics occur for any $\xi \in \Sigma _2$ since the fluid velocity is considered to be constant (i.e.,$f_2 (\xi ,\vartheta )=c$) throughout any cross-section of the channel perpendicular to the channel axis, which means that all particles in a given cross-section in $\Sigma _2$ have same velocity and direction of motion. Further, for all $\xi \in \Sigma _3$ the vector filed $f_3 (\xi ,\vartheta )=f_1 (\xi ,\vartheta )\mid _{h_{pl}=0}$.

The equilibrium points (or stagnation points) are the solution points $\bar{\xi }$ in a flow field where the local velocity $\dot{\xi }$ of the fluid found to be zero. The classification of equilibria into admissible, virtual, and boundary points is critical for capturing the system’s dynamic.

### Definition 1

Assume that $\mathcal {\check{P}}\in \mathbb {R}^2$ is a equilibrium point of the system (27), then

- If ${\check{\mathbb{P}}}\in \Sigma _i$ and $F_j\mid _{\check{\mathbb{P}}}=0$ for any $i=j$, $i,j=1,2,3$, then ${\check{\mathbb{P}}}$ is referred as an admissible(valid) point.
- If ${\check{\mathbb{P}}}\in \Sigma _i$ and $F_j\mid _{\check{\mathbb{P}}}=0$ for any $i\ne j$, $i,j=1,2,3$, then ${\check{\mathbb{P}}}$ is referred to as a virtual point (because it is not located in its associated region).
- If ${\check{\mathbb{P}}}:=\{x\in \mathbb {R}^2\mid y=h_{pl} ~ \text {and} ~ f_1({\check{\mathbb{P}}})=0\}$ or ${\check{\mathbb{P}}}:=\{x\in \mathbb {R}^2\mid y=0 ~ \text {and} ~ f_3({\check{\mathbb{P}}})=0\}$, then ${\check{\mathbb{P}}}$ is referred to a boundary point.

Switches between distinct types of dynamical behavior may be performed by describing the transition of virtual to admissible equilibrium points, as well as the possibility of bifurcation in each domain independently.

It is beneficial to consider a specific situation in order to fully comprehend the basic flow motion through the wavy channel. Hence, we consider the flow mode when $\acute{u_{e}}=0$ and therefore the two-dimensional flow is defined by stream function as:

28 $$\begin{aligned} \Psi _c&= \left\{ \begin{array}{cc} \frac{1}{6}\,\frac{\partial p_c}{ \partial x} \left( h-y \right) ^{2} \left( 2h+y-3h_{pl} \right) -y+h+q,&{} y\in \Sigma _1,\\ c y,&{} y\in \Sigma _2,\\ \frac{1}{6}\,\frac{\partial p_c}{ \partial x}\mid _{h_{pl}=0} \left( h-y \right) ^{2} \left( 2h+y \right) -y+h+q, ,&{} y\in \Sigma _3, \end{array} \right. \end{aligned}$$

where $\frac{\partial p_c}{\partial x}=\frac{-3\left( q+h-h_{pl}\right) }{ \left( h-h_{pl} \right) ^{3}}$. Hence, the vector fields of the dynamical system (27) is identified as:

29 $$\begin{aligned} f_1 (\xi ,\vartheta )=\left( \begin{array}{c} -\frac{1}{2}\frac{\partial p_c}{ \partial x}\, \left( h-y \right) \left( h-2\,h_{pl}+y \right) -1 \\ \frac{\partial h}{\partial x}\left( \frac{\partial p_c}{\partial x}\left( h-y\right) \left( h_{pl}-y-h\right) -1\right) +\\ \frac{1}{6}\frac{\partial ^2 p_c}{\partial x^2} \left( h-y \right) ^{2} \left( 3h_{pl} -2h-y \right) \\ \end{array} \right) , \,\, f_2 (\xi ,\vartheta )=\left( \begin{array}{c} c \\ 0 \\ \end{array} \right) , ~ f_3 (\xi ,\vartheta )=f_1 (\xi ,\vartheta )\mid _{h_{pl}=0}. \end{aligned}$$

The identification of equilibrium points and the study of their stability is an important step in the analysis of fluid dynamics. The equilibrium points of the vector fields (29) can be derived by setting $f_1 (\xi ,\vartheta )=0$ and $f_2 (\xi ,\vartheta )=0$. Then, by solving the obtained system of nonlinear equations, the equilibrium point are obtained as:

30 $$\begin{aligned} \bar{y}=\pm \frac{\bar{h}-h_{pl}}{\sqrt{3}} \sqrt{\frac{3q+\bar{h}-h_{pl}}{q+\bar{h}-h_{pl}}}+h_{pl} \in \Sigma _1,~~ \bar{y}=\pm \frac{\bar{h}}{\sqrt{3}} \sqrt{\frac{3q+\bar{h}}{q+\bar{h}}} \in \Sigma _3 \end{aligned}$$

where $\bar{h}$ is evaluated at the point $\bar{x}$. The values of $\bar{x}$ which represent the solutions of $\dot{x}=0$ is more complicated. One possible solution is given by setting $\frac{\partial h}{\partial x}=0$, i.e., $\bar{x}$ is the solution of

31 $$\begin{aligned} \sum _{i=1}^{m} 2 \pi i \varepsilon _i \sin {(2 \pi i x)}=0 \end{aligned}$$

For instance, let $m=2$, then the above equation becomes

$$\begin{aligned} \varepsilon _{{1}}\cos \left( 2\,\pi \,x \right) +2\,\varepsilon _{{2 }}\cos \left( 4\,\pi \,x \right) =0 \end{aligned}$$

which has a solution

$$\begin{aligned} \begin{aligned} \bar{x}&=\frac{1}{2 \pi }\,\arccos \left( {\frac{-\varepsilon _{{1}}+\sqrt{{\varepsilon _{{1}}}^{2}+ 32\,{\varepsilon _{{2}}}^{2}}}{8 \varepsilon _{{2}}}} \right) , \, \bar{x}=\frac{1}{2 \pi }\, \left( \pi +\arccos \left( \,{\frac{\varepsilon _{{1}}+\sqrt{{\phi _ {{1}}}^{2}+32\,{\varepsilon _{{2}}}^{2}}}{8 \varepsilon _{{2}}}} \right) \right) . \end{aligned} \end{aligned}$$

**Figure 2:**
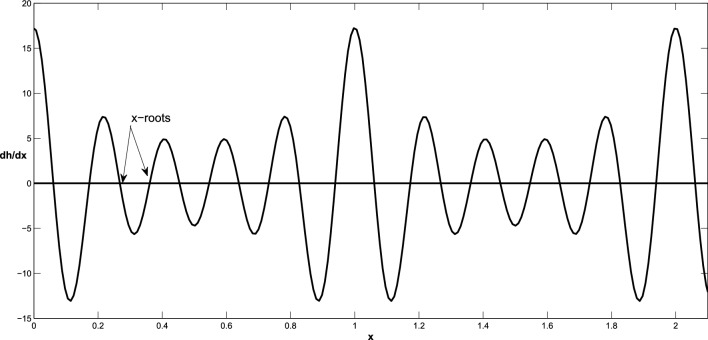
Nonlinear behavior of the function (31) and its x-roots.

**Figure 3:**
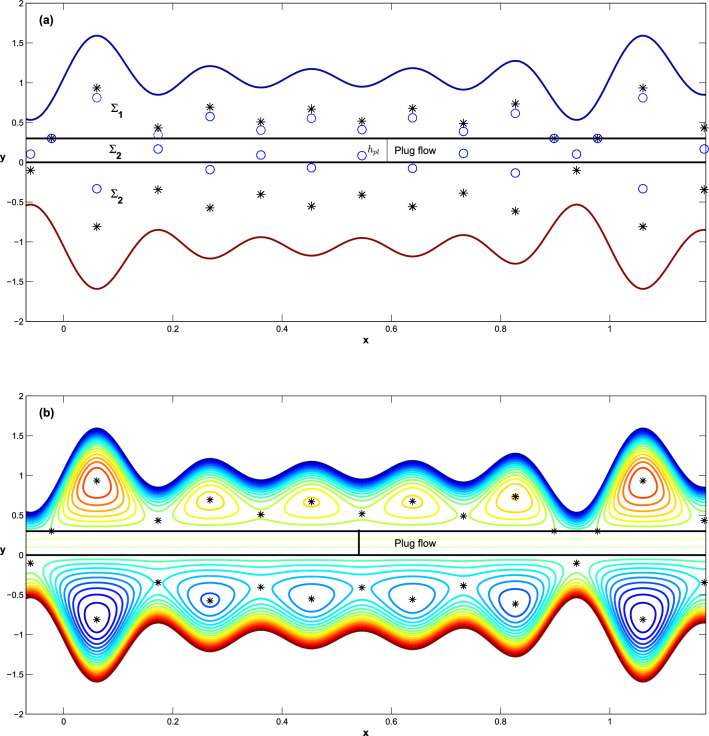
The location of equilibrium points, admissible point symbol $*$ and virtual point symbol $\circ$ and the dynamic behavior that surrounds admissible points, when $\acute{u_{e}}=0, h_{pl}=0.3, q=-0.123, \phi (j)=\Sigma _{j=1}^5 \frac{4 j}{100}$.

Note that if $m\ge 3$, it is difficult to find the zeros of the function (31) explicitly, we can compute the values of $\bar{x}$ using various numerical methods, such as the Newton method. Figure 2 shows that the nonlinear behavior of the function (31), whose roots are observed as x-intercepts, occurs at x-values where the function value is 0. It should be noted that the boundary points are deduced as $\mathbb {\check{P}}=\left( \bar{x},h_{pl}\right)$ and $\mathbb {\check{P}}=\left( \bar{x},0\right)$. Figure 3a illustrates the location of admissible and virtual equilibrium points in a specific situation.

The linearization of generalized system (27) at the point $\mathbb {\check{P}}=\left( \bar{x},\bar{y}\right) \in \Sigma _1$ ( or $\mathbb {\check{P}}\in \Sigma _3$) is given by

32 $$\begin{aligned} {\textbf{J}}=\left( \begin{array}{cc} \Psi _{xy} &{} \Psi _{yy} \\ -\Psi _{xx} &{} -\Psi _{yx} \\ \end{array} \right) _{\left( \bar{x},\bar{y}\right) }, \end{aligned}$$

such that if $\acute{u_{e}}= 0$, then $\Psi =\Psi _c$. We note that $trace(\textbf{J})=0$ and $\det (\textbf{J})= \Psi _{xx}\Psi _{yy} -\Psi _{yx}^2$ for all points $\left( \bar{x},\bar{y}\right) \in \mathbb {R}$. Thus, the equilibrium point $\mathbb {\check{P}}$ is either a saddle or a center for the Hamiltonian system (27) iff $\det (\textbf{J})<0$ or $\det (\textbf{J})>0$, respectively. If $\det (\textbf{J})=0$, the point $\mathbb {\check{P}}$ becomes degenerate equilibrium point.

The configuration of the possible bifurcation of the above explicit formulas of equilibrium point is sufficient to report the most of the significant different flow behavior that may occur in this situation. It is clear in Fig. 3b that the center points generate fluid boluses around them, and the saddle points connection in the context of the existing heteroclinic curve forms a trapping zone.

In general situation, $\acute{u_{e}}\ne 0$,

33 $$\begin{aligned} \Psi =&\left\{ \begin{array}{cc} {\acute{u_{e}} \frac{ \left( 0.5{D_{l}}^{2} \left( h-y \right) ^{2}\sinh \left( D_{l} h_{pl} \right) -\,D_{l} \left( h-y \right) \cosh \left( D_{l} h \right) +\sinh \left( D_{l} h \right) -\sinh \left( D_{l} y \right) \right) }{D_{l} \cosh \left( D_{l} h \right) }}+\Psi _c, &{} y\in \Sigma _1,\\ c y,&{} y\in \Sigma _2,\\ \Psi _c-{\acute{u_{e}} \frac{ \left( D_{l} \left( h-y \right) \cosh \left( D_{l} h \right) -\sinh \left( D_{l} h \right) +\sinh \left( D_{l} y \right) \right) }{ D_{l} \cosh \left( D_{l} h \right) }}, &{} y\in \Sigma _3, \end{array} \right. \end{aligned}$$

34 $$\begin{aligned} f_1 (\xi ,\vartheta )=&\left( \begin{array}{c} -\frac{1}{2}D_p\, \left( h-y \right) \left( h-2\,h_{pl}+y \right) -1+\acute{u_{e}} \Lambda _1 \\ \\ \frac{\partial h}{\partial x}\left( D_p\left( h-y\right) \left( h_{pl}-y-h\right) -1+u_{{e}}\Lambda _2\right) \\ + \frac{1}{6}\frac{\partial D_p}{\partial x} \left( h-y \right) ^{2} \left( 3h_{pl} -2h-y \right) \\ \end{array} \right) , \,\, f_2 (\xi ,\vartheta )=\left( \begin{array}{c} c \\ 0 \\ \end{array} \right) , ~ f_3 (\xi ,\vartheta )=f_1 (\xi ,\vartheta )\mid _{h_{pl}=0} \end{aligned}$$

where

$$\begin{aligned} \Lambda _{1}= & {} {\frac{ \left( D_{l} \left( y-h \right) \sinh \left( D_{l}{} { hp} \right) +\cosh \left( D_{l}h \right) -\cosh \left( D_{l}y \right) \right) }{ \cosh \left( D_{l}h \right) }},\\ \Lambda _2= & {} \,{\frac{ \left( \left( y-h \right) \left( D_{l} \left( h-y \right) \sinh \left( D_{l}h \right) -2\,\cosh \left( D_{l} h \right) \right) D_{l}\sinh \left( D_{l} h_{pl} \right) +2\,\sinh \left( D_{l} h \right) \left( \sinh \left( D_{l} y \right) -\sinh \left( D_{l} h \right) \right) \right) }{ 2 \left( \cosh \left( D_{l} h \right) \right) ^{2}}},\\ D_p= & {} \frac{\partial p_c}{\partial x}+3/2\,{\frac{\acute{u_{e}}\, \left( \left( 2- \left( h-h_{pl} \right) ^{ 2}{D_{l}}^{2} \right) \sinh \left( D_{l} h_{pl} \right) +2\,D_{l} \left( h-h_{pl} \right) \cosh \left( D_{l} h \right) -2\,\sinh \left( D_{l} h \right) \right) }{D_{l}\cosh \left( D_{l} h \right) \left( h-h_{pl} \right) ^{3}}}. \end{aligned}$$

We are unable to proceed with the explicit bifurcation analysis of the above vector fields due to their high level of nonlinearity. Therefore, in the following section, we will extend our analysis of bifurcation using an efficient numerical technique.

### Numerical bifurcation analysis

Numerical analysis is an important tool in dealing with nonlinear bifurcation problems in various biological and physical systems. One of the main advantage of this approach is that it is used to measure the steady-state curves for an undetermined system of equations.

The solutions to the following piecewise nonlinear system are helpful in determining all potentially admissible, virtual, boundary, and degenerate equilibrium points, as well as their bifurcation.

35 $$\begin{aligned} F\left( x,y,\vartheta \right) = {\left\{ \begin{array}{ll} f_1\left( x,y,\vartheta \right) =0, \quad \text {if } y\in \Sigma _1,\\ f_3(x,y,\vartheta )=0,\quad \text {if } y\in \Sigma _3,\\ \end{array}\right. } \end{aligned}$$

A nonlinear system $F\left( x,y,\vartheta \right) =0$ may have an infinite or finite number of roots, and these roots can be extremely sensitive to small changes in one or more parameters of $\vartheta$. Therefore, the main idea is to present an optimal technique for computing all roots of $F\left( x,y,\vartheta \right)$ and detecting various types of behavior around these roots, resulting in a complete picture of various multiphase (i.e. in $\Sigma _1$ and $\Sigma _3$) flow behaviors.

It should be noted that there is no equilibrium point for the system $\dot{\xi }=f_2\left( x,y,\vartheta \right) , y\in \Sigma _2$. However, some virtual points for the system (35) can be located in the domain of $\Sigma _2$ (see for example Fig. 3b). As a first stage in computing the equilibrium points, a fixed $\Pi$ is chosen to be a sector-like domain, which is defined as: $\Pi =\Pi _{1} \cup \Pi _{2}$ where $\Pi _1=\{\left( x,y\right) \in \mathbb {R}^2, \mid a \le x \le b, y\in \Sigma _1\}$ and $\Pi _2=\{\left( x,y\right) \in \mathbb {R}^2,\mid a\le x \le b, y\in \Sigma _3\}$. This implies that the sector $\Pi$ shears the same domain along the x-axis, weher $a,b\in \mathbb {Z}$. According to numerical domain decomposition, limiting the size of the solution domains improves computational efficiency and reduces the amount of computing effort necessary to solve the system (35). Then these real intervals of interest $\Pi _{1}$ and $\Pi _{2}$ will further divide into finite sub-intervals based on nonlinear system behaves at different regions (i. e., the sub-intervals are not necessarily equidistant) as: $\Pi _{1}=[\Pi _{1}^{\textbf{m}-1}, \Pi _{1}^{\textbf{m}}]$ and $\Pi _{2}=\left[ \Pi _{2}^{\textbf{n}-1}, \Pi _{2}^{\textbf{n}}\right] ,$ $\textbf{m}, \textbf{n} \in Z^+$. Then, in each subinterval $\Pi _{1}$ and $\Pi _{2}$, we use Newton’s iterative technique to solve the two system (35) independently, and the explicit equilibrium points (30) are considered an initial guess. Finally, we use the Matlab package (MatCont)for numerical bifurcation analysis to classify the output equilibrium points and calculate their linear stability and related bifurcation^39^).

**Figure 4:**
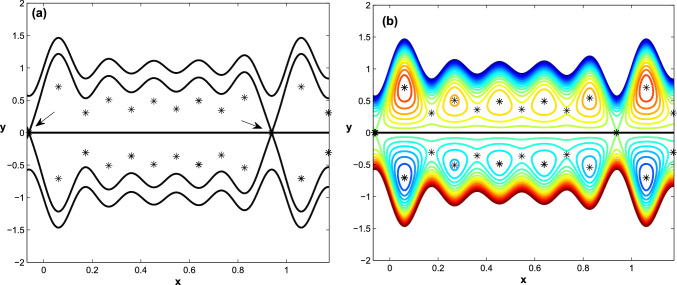
The maximum trapping zone occurs when $\acute{u_{e}}=D_{l}=1, h_{pl}=0.0, q=-0.18366$.

**Figure 5:**
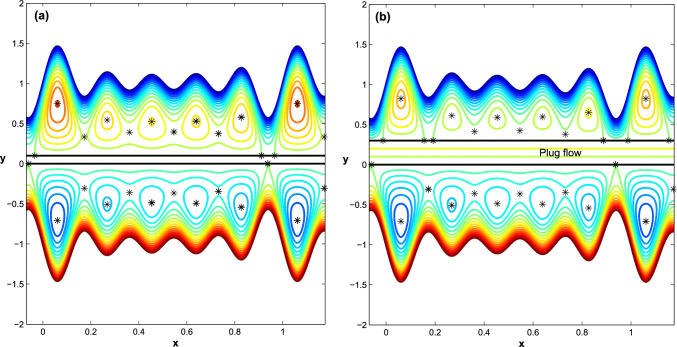
The impact of the parameter $h_{pl}$ on channel flow behavior in $\Sigma _1$, keeping all of the parameters with Fig. 4 fixed. (**a**) At $h_{pl}=0.1$, the heteroclinic connection between $\Sigma _1$ and $\Sigma _3$ is terminated. At $h_{pl}=0.3$, two distinct heteroclinic connections appear in $\Sigma _1$, and the trapping bolus size is reduced.

**Figure 6:**
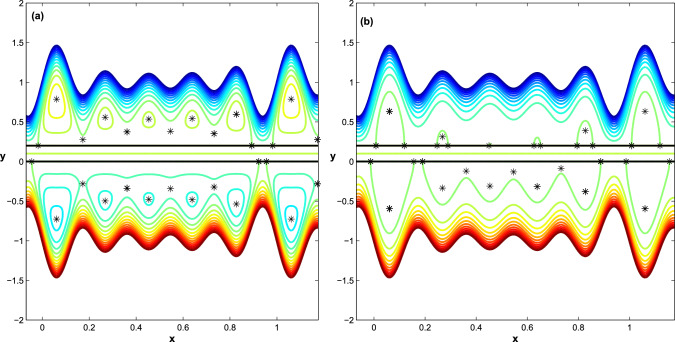
The effect of the Debye length parameter $D_{l}$ on trapped flow behavior ($\acute{u_{e}}=1, h_{pl}=0.2, q=-0.18366$): (**a**) $D_{l}=3$ and (**b**) $D_{l}=5$.

**Figure 7:**
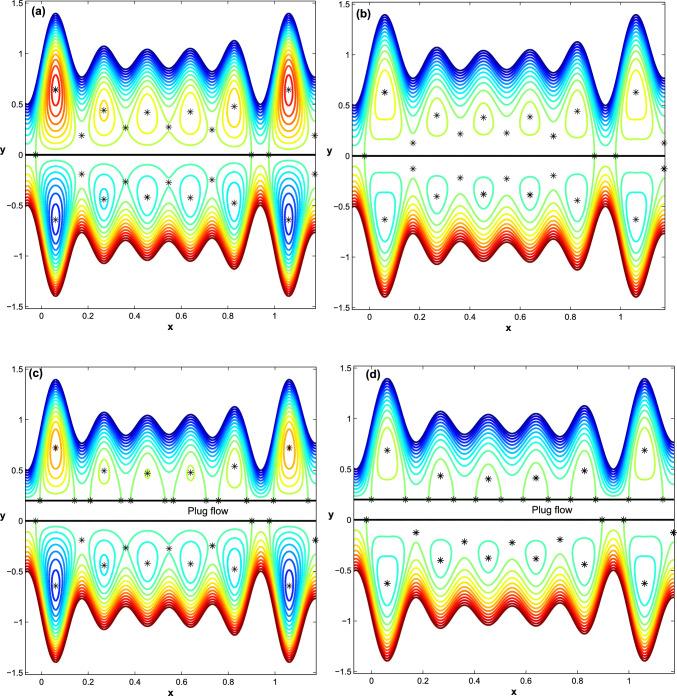
The effect of Helmholtz-Smoluchowski velocity $$\acute{u_{e}}$$ on trapped flow behavior ($$D_{l}=1.1, h_{pl}=0.0, q=-0.25 $$): at $$h_{pl}=0$$ (**a**)$$\acute{u_{e}}=-5$$ and (**b**)$$\acute{u_{e}}=5$$ and at $$h_{pl}=0.2$$ (**c**) $$\acute{u_{e}}=-5$$, (**d**) $$\acute{u_{e}}=5$$.

**Figure 8:**
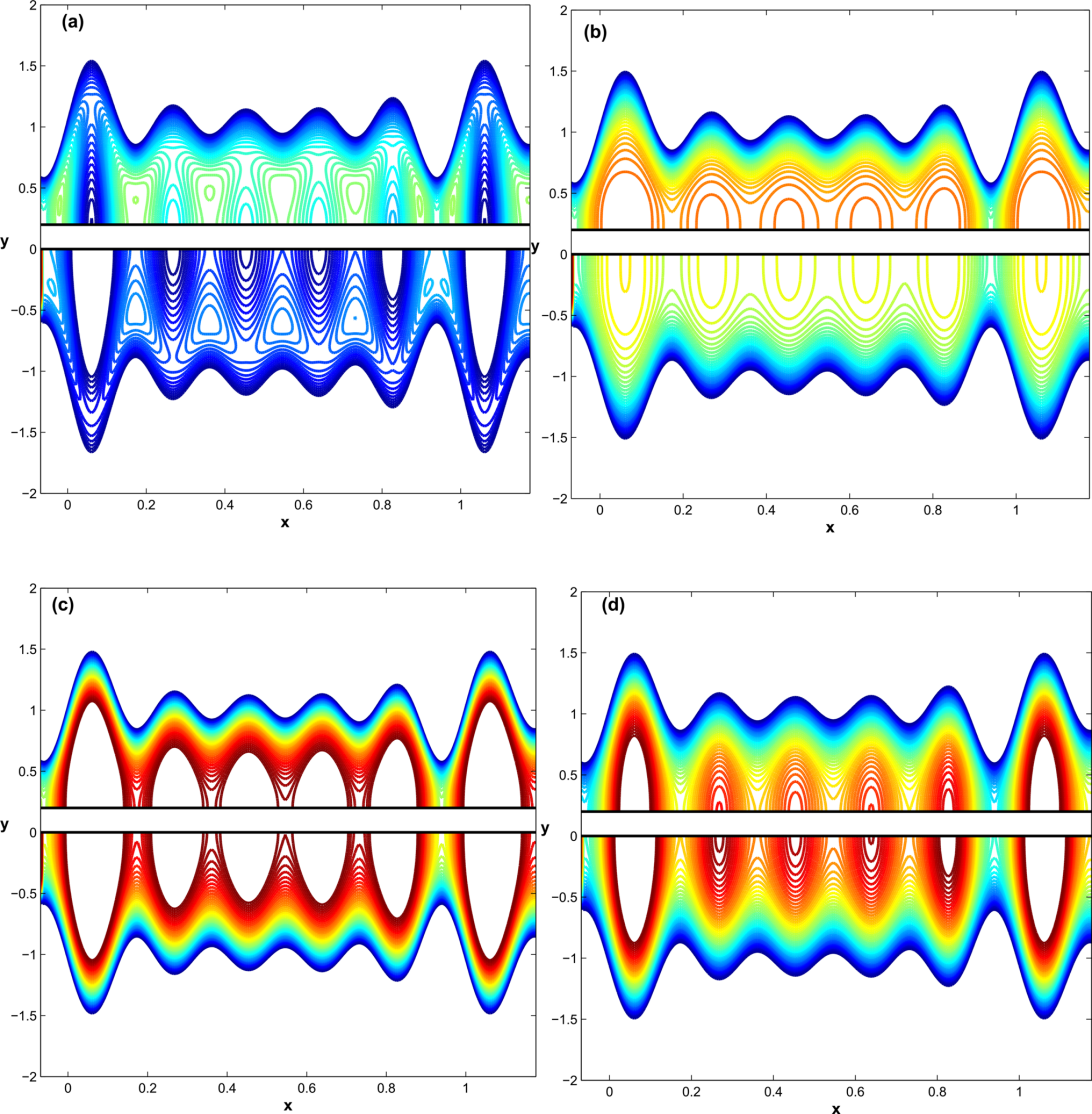
The contour of the full temperature field at $\acute{u_{e}}=D_{l}=1, h_{pl}=0.1, q=-0.18366$: for $Br=1.0$ and varying $\acute{G}$ as (**a**) $\acute{G}=-1$ (**b**) $\acute{G}=0.0$ (**c**) $\acute{G}=1$, whereas (**d**) holds for $\acute{G}=1.0$ and $Br=0.5$.

**Figure 9:**
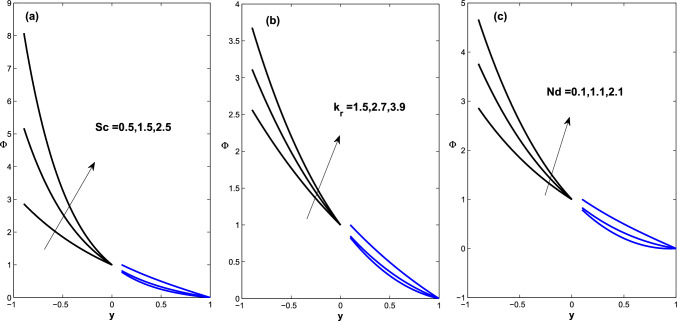
Fluid concentration behavior as a function of Schmidt number, chemical reaction, and concentration difference parameters: (**a**) $Sc =0.5,~1.25,~ 2.5$ (**b**) $k_r=1.2,~2.7,~ 2.9$, (**c**) $Nd =0.1,~1.1,~2.1$ .

## Results and discussion

In this section, we discuss the impact of different physical parameters on fluid flow behaviors using numerical bifurcation analysis.

Trapping phenomenon: Based on a dynamical examination of flow behavior around equilibrium points, this phenomenon can be discovered in two scenarios, namely local and global modes of system behavior. The flow is trapped in terms of local mode when the system (35) has center points (closed orbits) that cause the streamline to split to enclose a bolus of fluid particles. In general, the global mode is defined as structural changes in the characteristic frame that cannot be identified when examining the stability of equirbira by computing the eigenvalues of the associated Jacobian. A heteroclinic connection is a type of global mode formed when a streamline connects two or more saddle-points. One advantage of using heteroclinic connections in such a system is that the flow is completely trapped between the connection orbits. Furthermore, the distance between two saddle points for a heteroclinic connection is used to determine the minimum and maximum limits of the trapping zone. In all subsequent computations, we consider the amplitude of the various waves to be $\varepsilon =\Sigma _{j=1}^5 \frac{3.4 j}{100}$. In this context, Fig. 4 depicts the largest invariant curve connecting two distances of saddle points, which establishes the maximum trapping zone.

The plug flow region $\Sigma _2$ is identified and controlled by the parameter $h_{pl}$. Hence, we discuss how changes in the parameter $h_{pl}$ affect the flow behavior in the upper region of the channel due to changes in the location and stability of the equilibrium points. At $h_{pl}=0.1$ and keeping all the parameters fixed, as shown in Fig. 4. In Fig. 5a, we notice that the flow behavior in region $\Sigma _1$ changes due to the presence of a plug flow region and the heterocilinic connection between the upper and lower regions of the channel is destroyed, resulting in two distinct trapping regions, although the flow behavior in region $\Sigma _3$ remains unchanged. When the parameter $h_{pl}$ is increased to $h_{pl}=0.3$, two distinct heterocilinic connections emerge in the upper region, and the size of the trapping bolus is reduced, see Fig. 5b.

By taking the Debye length $D_{l}$ (i.e., the electrical double layer thickness) as a bifurcation parameter (all other parameters fixed as $\acute{u_{e}}=1, h_{pl}=0.2, q=-0.18366$), It is seen in Fig. 6 increasing the value of $D_{l}$ causes some equilibrium points to vanish (disappear), while others points move to become boundary points. The fixed parameters of the system are given as: $\acute{u_{e}}=1, h_{pl}=0.2, q=-0.18366$, where Fig. 6a is created with $D_l=3.0$ while Fig. 6b is created with $D_l=5.0$. This explains why the boluses are initially reduced in size and then vanish as the value of $D_{l}$ increases, i.e., the trapping zone does not occur with a large value of $D_{l}$.

Following that, we show how different Helmholtz-Smoluchowski velocity $\acute{u_{e}}$ variations affect flow behavior in the upper and lower regions of the channel in terms of bifurcation of equilibrium points. At $h_{pl}=0$, the channel becomes symmetric on either side of the *x*-axis. In addition, depending on whether the Helmholtz-Smoluchowski velocity parameter is negative or positive, the electrical field acts in the positive axial direction or is oriented in the reverse x-direction. The maximum trapping zone is shown in Fig. 7a and b, but the configuration of bolus dynamics inside this zone differs depending on whether $\acute{u_{e}}$ is positive or negative. When $\acute{u_{e}}=-5$, the size of boluses increases noticeably, whereas when $\acute{u_{e}}=5$, the size of boluses decreases. When $h_{pl}=0.2$, the flow in channel becomes asymmetric on both sides of the *x*-axis, see Fig. 7c and d. Further, the upper row of boluses converts to a family of heteroclinic connections that form a family of trapping zones that vary in size depending on whether $\acute{u_{e}}$ is negative or positive, whereas the lower row of boluses does not change.

Temperature fields in $\Sigma _1$ and $\Sigma _3$ are determined of an interesting situation when the flow is trapped in both regions using the same parameters as shown in Fig.5 (i.e., $\acute{u_{e}}=D_{l}=1, h_{pl}=0.2, q=-0.18366$). The contour of the temperature field across the microchannel is shown in Fig. 8a–c for negative, zero, and positive values of Joule heating term $\acute{G}$ and the Brinkman number is fixed $Br=1.0$ ( whereas Fig. 8d holds for $\acute{G}=1.0$ and $Br=0.5$) as the clarification of the changing and periodic temperature fields evolves to a nation of periodic dynamics. Fig. 9 depicts the fluid concentration behavior when the Schmidt number, chemical reaction, and concentration difference parameters are varied.

## Conclusions

The qualitative aspects of an electroosmotic peristaltic Bingham fluid model, specifically geometrical properties of flow fields such as bifurcation and the stability of its streamline patterns, are investigated. This model simulates the effect of heat and mass transfer on Bingham fluid flow through a complex wavy microchannel influenced by electroosmosis. The following are the key results of the current study:

- Analytical and numerical bifurcation analysis is used to systematically identify dynamic behavior and characterize fluid flow to reveal associated physical phenomena.
- Our results indicate that heteroclinic connections to saddle points are the primary cause of the trapping phenomenon in two scenarios, namely the presence or absence of symmetric flow.
- The non-uniform geometry caused by the plug region and varying amplitude ratio parameters has a significant impact on the trapping phenomenon.
- We assert that the trapping zone does not exist with a large Debye length $D_l$ because the model’s equilibrium points vanish (disappear) or become boundary points.
- The impact of Helmholtz-Smoluchowski velocity $\acute{u_{e}}$ has been demonstrated in a case with interesting and complex behavior. The flow, for example, generated a maximum trapping zone; our bifurcation findings explain why the configuration of the bolus dynamics within this zone varies depending on whether$\acute{u_{e}}$ is positive or negative.
- A parametric study is carried out to evaluate the effect of the Joule heating term, chemical reaction, and the Brinkman and Schmidt numbers on the temperature field contour across the microchannel and fluid concentration behavior.

## Data Availability

The datasets analysed during the current study are available from the corresponding author on reasonable request.

## List of symbols

$\acute{a}$, $\widehat{H}$, $\widehat{t}$, $\widehat{\varepsilon _{i}}$: The microchannel’s half width, transverse wall vibration of the microchannel, time, and amplitude of waves in fixed frame
$\lambda$, *c*, $\mu$, $\rho$, $\rho _{e}$: Wavelength, wave speed, dynamic viscosity of fluid, fluid density, density of the total ionic charge
$\widehat{S}_{x y}$, $\widehat{S}_{0}$, $\acute{S}$, $\gamma ^.$: Shear stress, yield stress, viscous dissipation, and rate of shear strain
$\widehat{U},\widehat{V}$, $\widehat{P}$, $\widehat{E_{x}}$, $\widehat{E_{y}}$: Velocity components, pressure in fixed frame, components of electric field $\widehat{E}$, respectively
$C_{p}$, *K*, $K_{\acute{r}}$, ${\sigma }$: Specific heat, thermal conductivity, rate of chemical reaction on species concentration, and electrical conductivity of the fluid
$\widehat{T}$, $\widehat{C}$, $\Theta$, $\acute{u}_{e}$, $\acute{G}$: Dimensional temperature, Concentration, electrical potential function, Helmholtz-Smoluchowski velocity, and normalized Joule heating term
$\acute{\varepsilon }$, $\widehat{n}_{+}$, $\widehat{n}_{-}$, *ze*: Electrical permittivity of ionic solution, positive and negative ions with the bulk concentration $\widehat{n}_0$, and *z* is the valency of ions, *e* denotes the electron charge
$D_{m}$, $k_{B}$, $T_{a}$: Diffusivity of chemical species, Boltzmann constant, and average or mean temperature of electrolytic solution
$T_{e}$, $T_{s}$, $C_{e}$, $C_{s}$: Internal temperature of the fluid, surface temperature, internal concentration of the fluid, and surface concentration, respectively
$D_{l}$, ${k}_{r}$, *Nd*: Debye length, chemical reaction, and concentration difference parameters
*Re*, *Br*, *Sc*: Reynolds, Brinkman, and Schmidt numbers, respectively
